# Dynamics and Function of Foliar Endophytic Bacterial Communities of *Ammopiptanthus mongolicus* Across Different Leaf Growth Stages

**DOI:** 10.3390/plants15020240

**Published:** 2026-01-13

**Authors:** Xue Wu, Yu Liao, Manmei Wu, Rui Yang, Qing Ma, Yuchen Wei, Jianli Liu

**Affiliations:** School of Biological Science and Engineering, North Minzu University, Key Laboratory of Ecological Protection of Agro-Pastoral Ecotones in the Yellow River Basin, National Ethnic Affairs Commission of the People’s Republic of China, Ningxia Key Laboratory of Microbial Resources Development and Applications in Special Environment, Yinchuan 750021, China; zstldxy@163.com (X.W.); camellialiyu@126.com (Y.L.); mmeiwu@163.com (M.W.); m18795087952@163.com (R.Y.); 15597451653@139.com (Q.M.); 15994340087@163.com (Y.W.)

**Keywords:** leaf, endophyte, bacterial community, diversity, Illumina Miseq, *Ammopiptanthus mongolicus*

## Abstract

*Ammopiptanthus mongolicus* is a relict species from the ancient Mediterranean of the Tertiary period and the only strong xerophytic evergreen broad-leaved shrub in the central Asian desert. Foliar endophytic and epiphytic bacteria jointly form phyllosphere microorganisms that influence plant health. This study investigated the dynamic changes in foliar endophytic bacterial communities across four leaf growth stages (Young, Mature, Old1, and Old2). Illumina 16S region (V5–V7) amplicon sequencing was used to analyze community composition, function, construction process, and environmental driving factors. The Old1 and Old2 stages were clearly separated from the Young and Mature stages, which demonstrated closer clustering. Community diversity and evenness first increased from the Young to Mature stages, declined at the Old1 stage, and finally reached maximum values at the Old2 stage; richness increased gradually. Total amplicon sequence variant (ASV) numbers, stage-specific ASVs, and their proportion increased with leaf development, whereas the proportion of shared ASVs between adjacent, interval, and all stages decreased. Dominant genera were *Rhodococcus* (Young), unclassified_f__Comamonadaceae (Mature), *Rhodococcus* (Old1), and *Bacillus* (Old2). Co-occurrence networks became progressively simpler, with reduced inter-node and positive connectivity. Functional predictions revealed that chemoheterotrophy and aerobic chemoheterotrophy decreased initially and then increased, with the lowest values at Old1. N, C/P, N/P, and SOD reached maximum at the Old2 stage. P was maximum at the Mature stage. P, C/P, and N/P were significantly positively correlated with the Young stage, N with the Mature stage, and SOD with the Old2 stage (*p* < 0.05). These findings enhance understanding of the diversity, composition, function, and plant–endophyte relationships in xerophytic relict species, particularly evergreen desert shrubs.

## 1. Introduction

*Ammopiptanthus mongolicus* (Maxim. ex Kom.) Cheng f., belonging to the genus *Ammopiptanthus* in the Fabaceae family, is a relict species from the ancient Mediterranean during the Tertiary period and holds significant scientific value for studying the origin and formation of steppe deserts in central Asia [1]. It is the only evergreen broad-leaved small shrub with strong xerophytic traits in the desert region of central Asia. The species exhibits high tolerance to drought, salinity–alkalinity, cold, heat, wind, and sand erosion, as well as other stresses [2]. The species functions as an edificator in the fragile West Ordos desert ecosystem and plays a key role in the stability and maintenance of steppe desert ecosystems [3,4].

Endophytic bacteria inhabit intercellular spaces or reside within the cells of various tissues and organs of healthy plants at specific stages or throughout the life cycle. Their presence is confirmed by isolation from strictly surface-disinfected plant tissues or by direct PCR amplification of microbial DNA from plant tissues [5,6]. As important members of the plant microbial community, endophytic bacteria maintain close associations with host plants. Certain endophytic bacteria promote plant growth [7,8,9], prevent and control plant diseases and pests [10,11,12,13], enhance plant stress resistance [14,15], and even certain taxa serve as a nitrogen source for plant growth under particular conditions [16,17]. Leaves, as essential organs for photosynthesis, harbor both endophytic and epiphytic bacteria, which together form phyllosphere microorganisms involved in photosynthesis, transpiration [18], stress tolerance [19], litter decomposition [20], and resistance to foliar pests and diseases [21,22]. Research on foliar endophytes is receiving growing attention. However, as *A. mongolicus* is the only evergreen broad-leaved shrub in the West Ordos desert ecosystem and a relict species from the ancient Mediterranean, this is the first study to examine stage-wise foliar endophyte dynamics in the species.

This study employed amplicon sequencing to analyze the endophytic bacterial communities in *A. mongolicus* leaves at different growth stages and assess how their dynamics respond to leaf development. The investigation further examined the effects of host tissue physiological state, reflected by physiological and biochemical indicators associated with stress tolerance, and the available nutrients in the endophyte survival microenvironment, represented by carbon, nitrogen, and phosphorus stoichiometry, on leaf endophytic bacterial communities. The study also identified the key factors shaping the foliar endophytic bacterial community structure in *A. mongolicus*.

## 2. Materials and Methods

### 2.1. Study Site

The study area is located in Mengxi Town, Etuoke County, Ordos City, Inner Mongolia Autonomous Region (106°53′5″ to 106°53′31″ E, 40°4′34″ to 40°4′28″ N), at an altitude of 1100–1200 m. The region is a typical steppified desert with a temperate continental climate and a mean annual temperature of 6–7 °C. The mean annual sunshine duration is approximately 2800 h, and the mean annual precipitation is about 200 mm, mainly concentrated from July to September. Mean annual evaporation is 2500–2700 mm, with the highest values from May to July. The predominant soil types are brown calcareous soil and gray desert soil [2].

Five *A. mongolicus* communities (20 m × 20 m) with the species as the dominant vegetative type were selected as sample plots, each spaced 100 m apart. The main associated species included *Zygophyllum xanthoxylum*, *Nitraria tangutorum*, *Reaumuria songarica*, *Allium mongolicum*, *Bassia dasyphylla*, and *Corispermum hyssopifolium*.

### 2.2. Leaf Sampling

**Figure 1:**
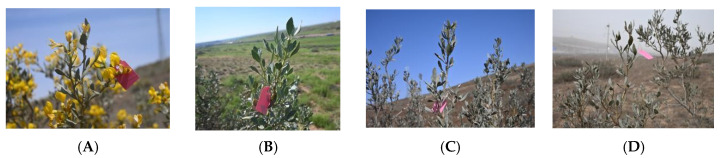
Leaf morphology of *A. mongolicus* at different growth stages. (**A**) young leaf stage (Young); (**B**) full leaf stage (Mature); (**C**) old leaf stage I (Old1); and (**D**) old leaf stage II (Old2).

In each sample plot containing six to ten *A. mongolicus* plants, five uniformly growing plants were randomly selected. One well-developed branch from each plant was collected from the east, south, west, and north orientations. From the top of each branch, six to eight healthy leaves were collected and combined as the sample for the respective plot. The samples were stored in a low-temperature preservation container and transported to the laboratory. Sampling covered a complete leaf growth and development cycle of the same trees, which were tagged with plastic labels. A total of 20 samples were collected on 4 May 2024 (young leaf stage, Young), 5 August 2024 (full leaf stage, Mature), 10 December 2024 (old leaf stage I, Old1), and 6 March 2025 (old leaf stage II, Old2) (Figure 1). Each sample was divided into two portions: one portion was surface-sterilized and stored at −80 °C for sequencing and determination of enzyme activity related to plant stress tolerance; the other portion was oven-dried for stoichiometric analysis.

### 2.3. Stoichiometric Characteristics of Carbon, Nitrogen, and Phosphorus

Collected leaves were oven-dried at 70 °C to a constant weight. The samples were ground into powder using a ball mill for the determination of stoichiometric characteristics. Leaf C content was determined using the potassium dichromate external heating method; phosphorus (P) content was determined using the acid digestion–molybdenum–antimony colorimetric method; and nitrogen (N) content was determined using the Kjeldahl method [23,24].

### 2.4. Physiological and Biochemical Characteristics Related to Plant Stress Tolerance

Soluble sugar (SC) content was determined using the Plant Soluble Sugar Content Kit (visible spectrophotometry, cat#KT-2-Y, detection limits 1 mg/g raw weight). Proline (PRO) content was determined using the Proline Content Kit (visible spectrophotometry, cat#PRO-2-Y, detection limits 1 μg/g raw weight); catalase (CAT) activity was determined using the catalase kit (ammonium molybdate colorimetric method, cat#CAT-2-W, detection limits 1 μmol/min·g raw weight); superoxide dismutase (SOD) activity was determined using the SOD kit (WST-8 method, cat#SOD-2-W, detection limits 1 U/g raw weight); peroxidase (POD) activity was determined using the POD kit (visible spectrophotometry, cat#POD-2-Y, detection limits 1 U/g raw weight); and malondialdehyde (MDA) content was determined using the MDA test kit (visible spectrophotometry, cat#MDA-2-Y, detection limits 1 nmol/g raw weight). All test kits were purchased from Suzhou Keming Biotechnology Co., Ltd., Suzhou, Jiangsu, China.

### 2.5. Extraction of DNA, Library Preparation, and Illumina MiSeq Sequencing

Leaves were rinsed with sterile running water to remove surface debris, followed by sequential sterilization with 75% ethanol and sodium hypochlorite solution (0.5% final available chlorine) for 5 min and 3 min, respectively. The samples were washed five times with sterile water and dried using sterile filter paper. To verify sterilization success, 100 μL of the final rinse water was inoculated onto nutrient agar (NA) medium and cultured at 25 °C for 7 days. No visible colony growth was observed after 3 days [3,7]. 2 µL of the final rinse water was templated to PCR. No products were checked.

Total DNA was extracted using the Plant Genomic DNA Kit (DP305, Tiangen Biotech Co., Ltd., Beijing, China). DNA concentration and purity were assessed via 1% agarose gel electrophoresis. Nested PCR amplification of the V5–V6–V7 regions of the bacterial 16S rRNA gene was performed using TransStart FastPfu DNA Polymerase (TransGen Biotech Co., Ltd., Beijing, China). First-round amplification used primer pair 799F (5′-AACMGGATTAGATACCCKG-3′) [25] and 1392R (5′-ACGGGCGGTGTGTRC-3′) [26]; second-round amplification used primer pair 799F and 1193R (5′-ACGTCATCCCCACCTTCC-3′) [27]. The PCR reaction mixture (20 μL) contained 10 μL 2× Pro Taq, 10 ng DNA template, 0.8 μL each of forward and reverse primers (5 μmol/L), and ddH_2_O to 20 μL. PCR conditions: 95 °C for 3 min (pre-denaturation), followed by 35 cycles of 95 °C for 30 s (denaturation), 55 °C for 30 s (annealing), and 72 °C for 45 s (extension), and final extension at 72 °C for 10 min. PCR products were verified by 2% agarose gel electrophoresis, recovered, and purified using the AxyPrep DNA Gel Extraction Kit (Axygen Biosciences, Union City, CA, USA). Quantification was performed using a Quantus Fluorometer (Promega Corporation, Madison, WI, USA). Libraries were constructed using the Illumina DNA Prep Kit (Illumina, San Diego, CA, USA) and sequenced on the Illumina MiSeq PE250 platform (Illumina, San Diego, CA, USA). DNA extraction, PCR amplification, and library construction were completed by Majorbio Bio-Pharm Technology Co., Ltd. (Shanghai, China). Sequence data were deposited in the NCBI Sequence Read Archive under accession number PRJNA1219371.

### 2.6. Bioinformatics and Statistical Analyses

After filtering, trimming, merging paired reads, removing chimera (parameters: truncLen = 0, maxEE = c(3,3), truncQ = 2, maxN = 0, trimLeft = c(17,20), minLen = 200, rm.phix = TRUE), qualified sequences were assigned to amplicon sequence variants (ASVs) using DADA2 software [28]. ASVs were taxonomically classified and annotated using the SILVA database (v138.1) [29]. Bioinformatic analysis was conducted using the Majorbio Cloud platform (https://cloud.majorbio.com) [30]. Statistical significance of alpha diversity, differential taxa, predicted functions among multiple groups was assessed using the Kruskal–Wallis test with post hoc test using Welch’s test. *p*-values were corrected for multiple testing using the false discovery rate (FDR).

Analysis of the assembly process of the community was conducted using the icamp package (version 1.5.12) in R (version 3.3.1) with β-NTI (Beta-Nearest Taxon Index). The randomization number was 99.

## 3. Results

### 3.1. Diversity of Foliar Endophytic Bacterial Communities of A. mongolicus at Different Leaf Growth Stages

The α-diversity indices of foliar endophytic bacterial communities varied across leaf growth stages. Community richness indices (Sobs, Chao, and Ace) increased progressively and reached their maximum at the Old2 stage, with values of 244.60, 245.47, and 245.50, respectively. These values were approximately 2.53 times higher than those of the Young stage. Diversity indices (Shannon and Simpson) and the evenness index (Pielou) increased significantly from the Young to Mature stages, decreased at the Old1 stage, and then rose to their highest levels at the Old2 stage. The Simpson and Pielou values were highest at the Mature and Old2 stages. The Simpson values for the Mature and Old2 stages were 0.95 and 0.97, respectively, representing 1.27-fold and 1.50-fold increases compared with the Young and Old1 stages. Pielou values for the Mature and Old2 stages were 0.82 and 0.80, representing 1.40-fold and 1.60-fold increases relative to the Young and Old1 stages, respectively. The highest Shannon index was recorded at the Old2 stage (4.37), followed by the Mature stage (3.87). No significant differences were observed in community coverage across growth stages. Richness increased significantly at the Old1 and Old2 stages, whereas diversity and evenness increased initially, decreased, and then increased again with leaf development (Table 1).

**Figure 2:**
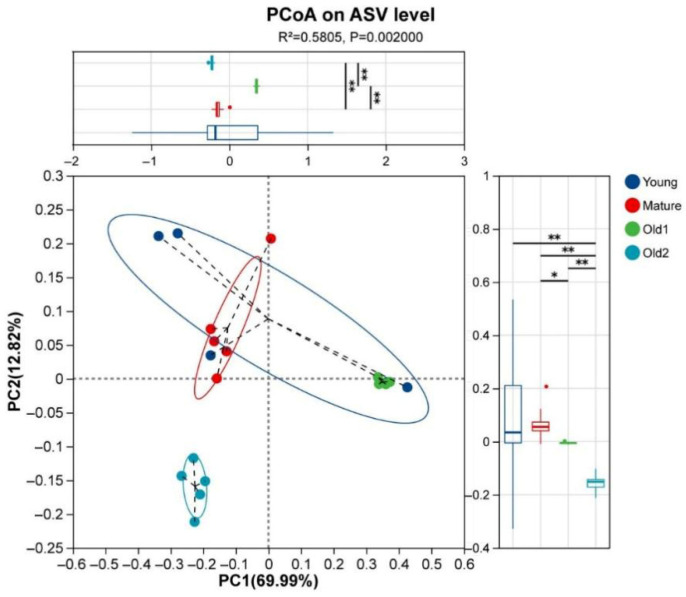
PCoA based on weighted UniFrac distances for foliar endophytic bacterial communities of *A. mongolicus* at different leaf growth stages at the ASV level. Young, foliar endophytic bacterial community at the young leaf stage; Mature, mature leaf stage; Old1, old leaf stage I; and Old2, old leaf stage II. Asterisks denote significance: * 0.01 < *p* ≤ 0.05; ** 0.001 < *p* ≤ 0.01.

Foliar endophytic bacterial communities were clustered using weighted UniFrac distance based on ASVs. The samples from the four growth stages were clearly separated. ANOSIM indicated significant intergroup differences (R = 0.5773, *p* = 0.001). PERMANOVA also showed relatively high intergroup variation (R^2^ = 0.5805, *p* = 0.002). The Young and Mature stage samples exhibited greater dispersion than the Old1 and Old2 samples, with the Young samples showing the highest dispersion. The Old1 stage samples were separated from the Mature and Old2 samples on the PC1 axis, whereas the Old2 samples were distinguished from the Mature and Old1 samples on the PC2 axis (Figure 2).

### 3.2. Composition of Foliar Endophytic Bacterial Communities of A. mongolicus at Different Leaf Growth Stages

**Figure 3:**
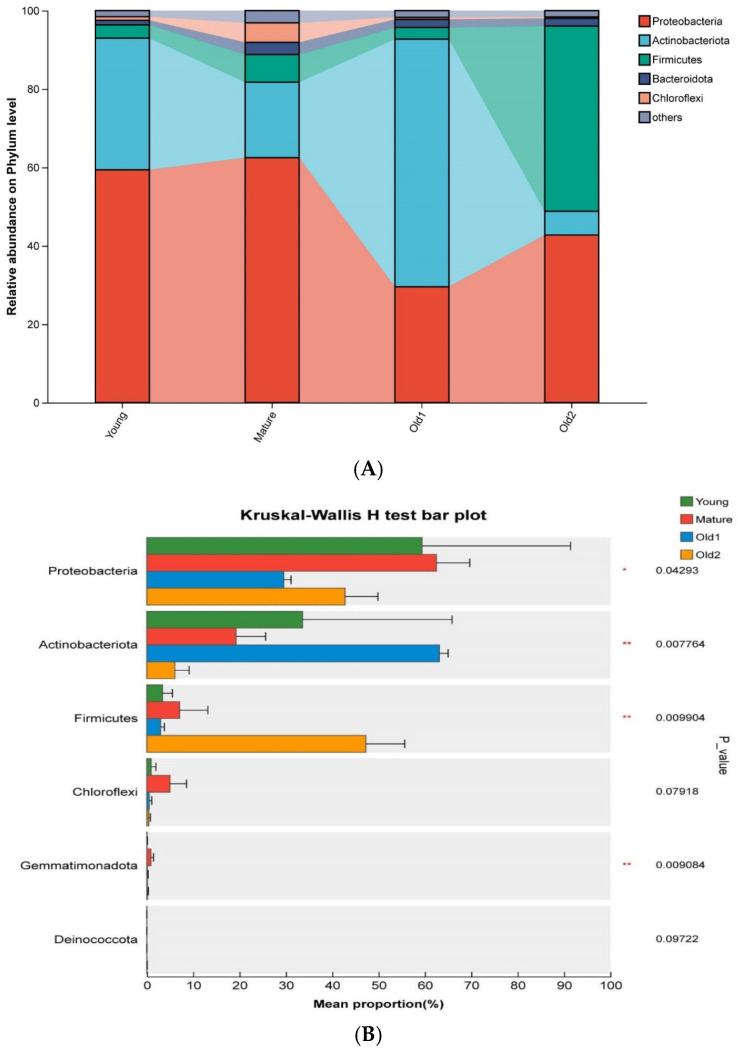
Relative abundance and statistical differences of foliar endophytic bacterial communities of *A. mongolicus* at different leaf growth stages at the phylum level. (**A**) Relative abundance of phyla; (**B**) differences in phylum-level relative abundance. Asterisks denote significance: * 0.01 < *p* ≤ 0.05; ** 0.001 < *p* ≤ 0.01.

A total of five phyla were identified in foliar endophytic bacterial samples across four leaf growth stages. The dominant phyla varied with leaf development. Proteobacteria were dominant overall, followed by Actinobacteriota and Firmicutes in the Young and Mature stage samples, respectively. Actinobacteriota dominated in the Old1 stage samples, whereas Firmicutes were dominant in the Old2 stage samples (Figure 3A). Differential analysis showed that Proteobacteria, Actinobacteriota, Firmicutes, and Chloroflexi exhibited significant differences across growth stages. Proteobacteria initially showed no significant variation, and then decreased and later increased, with the highest abundance at the Young and Mature stages and the lowest at the Old1 stage. Actinobacteriota decreased initially, and then increased before declining again, with the peak abundance at the Old1 stage and the lowest abundance at the Old2 stage. Firmicutes showed no significant variation during the first three stages but increased significantly at the Old2 stage (Figure 3B).

At the family level, dominant taxa differed across leaf growth stages. In the Young stage, the dominant family was Nocardiaceae (25.67%), followed by Sphingomonadaceae (21.09%), Comamonadaceae (12.54%), and Burkholderiaceae (10.71%). In the Mature stage, Comamonadaceae was dominant (14.98%), with Burkholderiaceae as the second most abundant family (12.92%). In the Old1 stage, *Nocardiaceae* was the dominant taxon (58.67%), followed by Comamonadaceae (8.20%) and Burkholderiaceae (6.27%). In the Old2 stage, Bacillaceae dominated (37.91%), followed by Comamonadaceae (15.17%) and Burkholderiaceae (11.14%) (Figure S1A). Differential analysis among the top 10 families showed significant differences in the relative abundance of Nocardiaceae, Bacillaceae, Beijerinckiaceae, and Planococcaceae. Nocardiaceae decreased initially, and then increased before declining again, with peak abundance at the Old1 stage. Bacillaceae showed no significant changes in the first three stages but increased significantly at the Old2 stage (Figure S1B).

**Figure 4:**
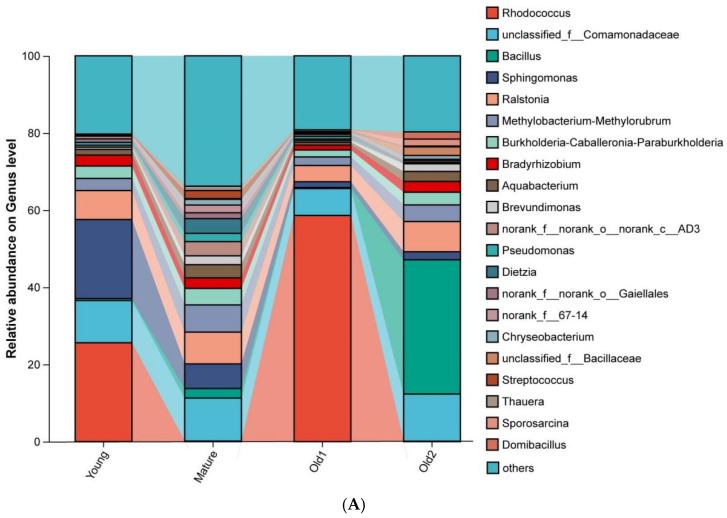

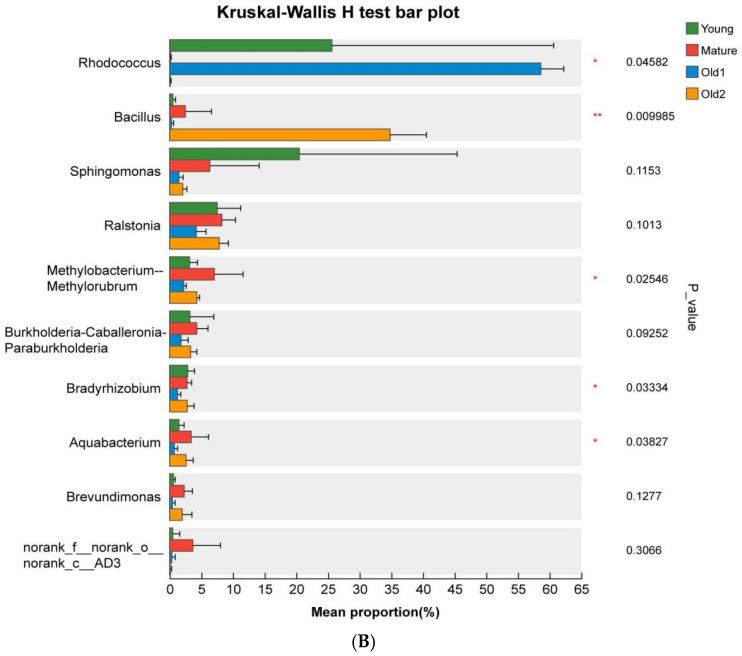
Relative abundance and statistical differences of foliar endophytic bacterial communities of *A. mongolicus* at different leaf growth stages at the genus level. (**A**) Relative abundance of genera; (**B**) differences in the relative abundance of genera. Asterisks denote significance: * 0.01 < *p* ≤ 0.05; ** 0.001 < *p* ≤ 0.01.

At the genus level, dominant genera of foliar endophytic bacterial communities varied across the four leaf growth stages of *A. mongolicus*. In the Young stage samples, the dominant genus was *Rhodococcus* (25.62%), followed by *Sphingomonas* (20.48%), unclassified_f__Comamonadaceae (10.95%), and *Ralstonia* (7.52%). In the Mature stage samples, unclassified_f__Comamonadaceae was dominant (11.15%), followed by *Ralstonia* (8.25%), *Methylobacterium–Methylorubrum* (7.05%), and *Sphingomonas* (6.36%). In the Old1 stage samples, *Rhodococcus* was dominant (58.61%), followed by unclassified_f__Comamonadaceae (6.98%) and *Ralstonia* (4.22%). In the Old2 stage samples, *Bacillus* was dominant (34.82%), followed by unclassified_f__Comamonadaceae (12.23%) and *Ralstonia* (7.83%) (Figure 4A). Difference analysis indicated significant variation in the relative abundance of *Rhodococcus*, *Bacillus*, *Beijerinckiaceae*, *Methylobacterium–Methylorubrum*, *Bradyrhizobium*, and *Aquabacterium* among the top 10 genera. *Rhodococcus* initially decreased, and then increased and decreased again, with peak abundance at the Old1 stage. *Bacillus* showed no significant change across the first three stages but increased significantly at the Old2 stage (Figure 4B).

LEfSe (Linear discriminant analysis (LDA) Effect Size) analysis using an LDA threshold of ≥3.5 identified *Bradyrhizobium* and unclassified_f__Rhodanobacteraceae as biomarkers for the Young stage. *Hydrogenophilus*, *Aquabacterium*, *Acidibacter*, *Conexibacter*, *Methylobacterium–Methylorubrum*, norank_f__67-14, norank_f__Gemmatimonadaceae, and unclassified_f__Xanthomonadaceae were biomarkers for the Mature stage. *Rhodococcus*, norank_f__Micropepsaceae, and *Methylotenera* were biomarkers for the Old1 stage. *Bacillus*, *Oceanobacillus*, unclassified_f__Bacillaceae, *Paenibacillus*, and unclassified_f__Planococcaceae were biomarkers for the Old2 stage (Figure S2).

**Figure 5:**
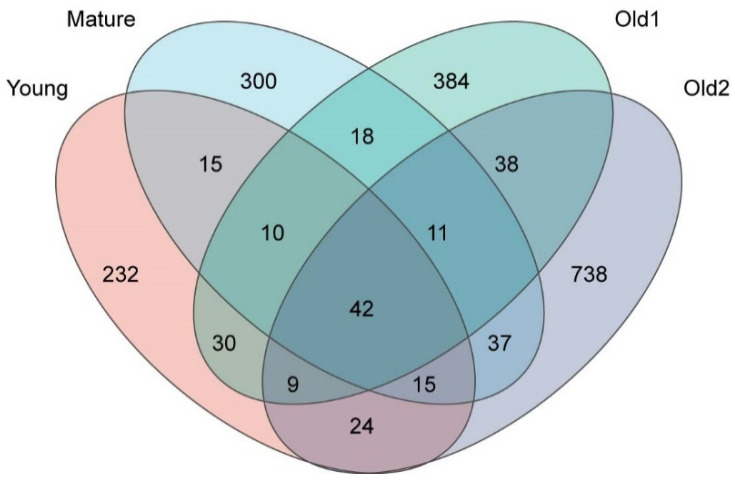
Venn diagram of foliar endophytic bacterial communities of *A. mongolicus* at different leaf growth stages at the ASV level.

At the ASV level, a total of 2281 ASVs were detected across the four leaf growth stages. The ASV count increased progressively with leaf development, with the Old2 stage (914) exhibiting 2.42 times the number recorded at the Young stage (377). The number of stage-specific ASVs also increased with leaf development, with the Old2 stage (738) exhibiting 3.16 times the number in the Young stage (232). The percentage of specific ASVs increased from 61.54% at the Young stage to 80.74% at the Old2 stage with leaf development. The number of common ASVs shared between two adjacent growth stages increased with leaf development, whereas their percentage decreased. The Young and Mature stage samples shared 82 common ASVs, accounting for 21.75% of the Young stage and 18.30% of the Mature stage samples. The Mature and Old1 stage samples shared 81 ASVs, accounting for 18.08% and 14.94% of the Mature and Old1 stage samples, respectively. The Old1 and Old2 stage samples shared 100 ASVs, accounting for 18.45% of the Old1 stage and 10.94% of the Old2 stage samples. The percentage of ASVs common to all four growth stages decreased progressively. A total of 42 ASVs were shared across all stages, accounting for 11.14% of the Young, 9.38% of the Mature, 7.75% of the Old1, and 4.59% of the Old2 stage samples (Figure 5). Overall, the total ASV count, number of unique ASVs, and their proportion increased with leaf development, whereas the percentage of shared ASVs between adjacent stages, two-interval stages, and across all four stages decreased.

### 3.3. Co-Occurrence Networks of Foliar Endophytic Bacterial Communities of A. mongolicus at Different Leaf Growth Stages

**Figure 6:**
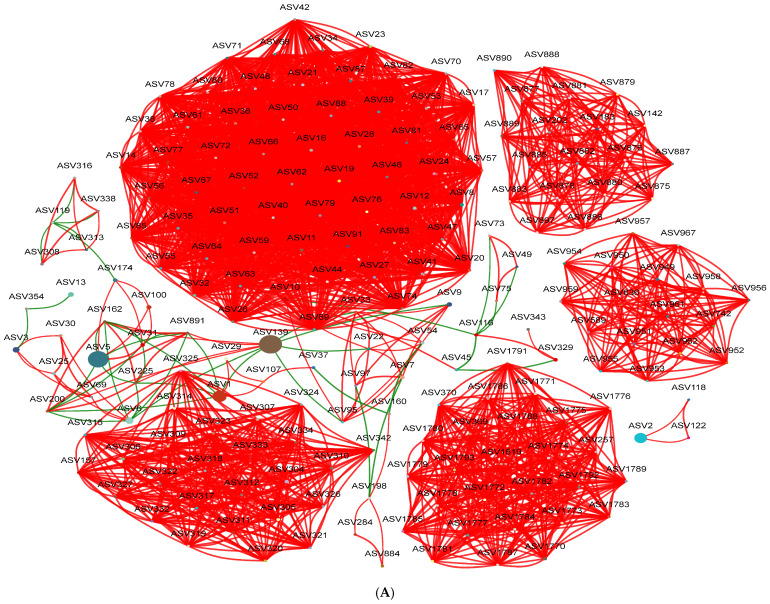

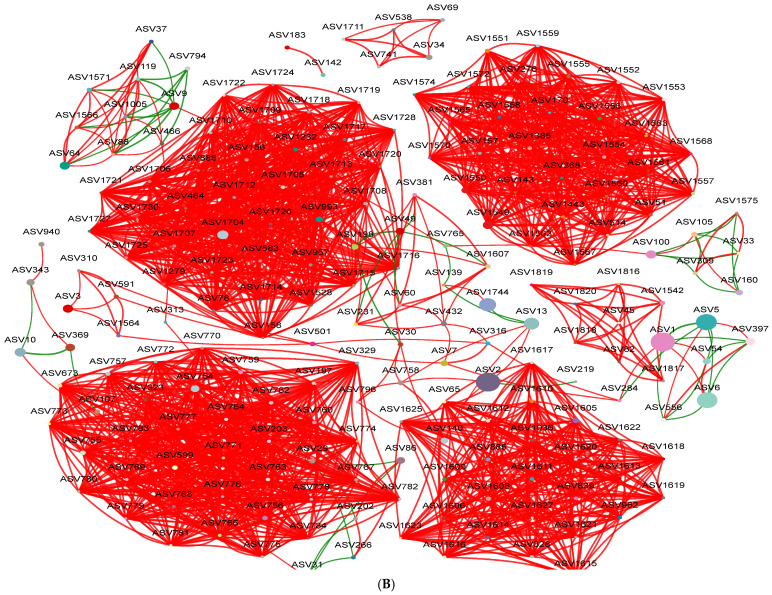

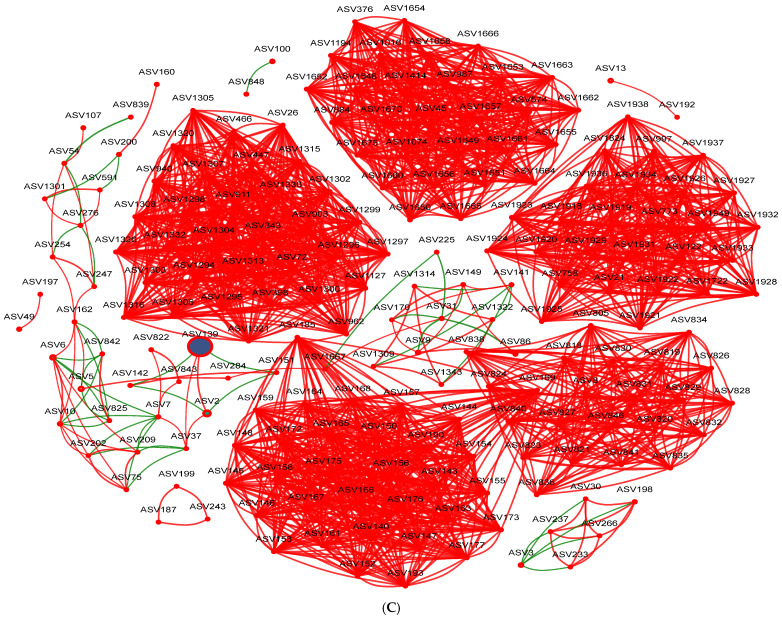

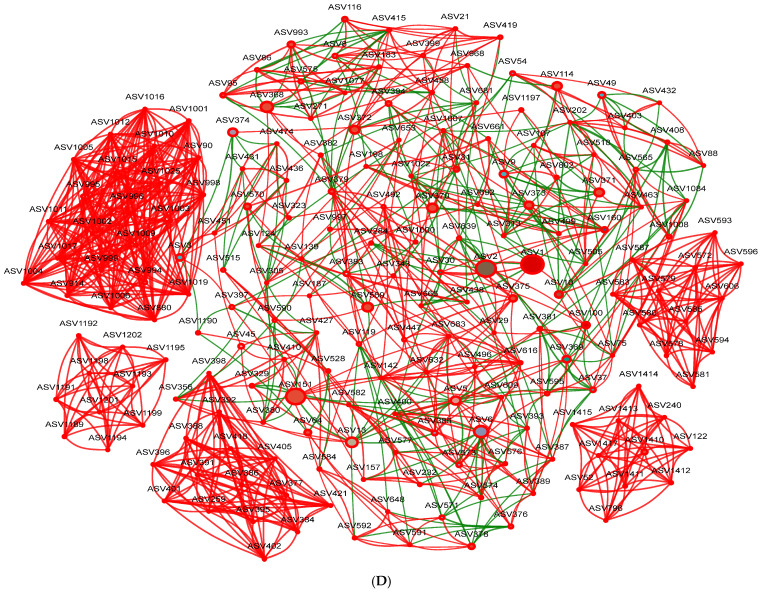
Co-occurrence networks of foliar endophytic bacterial communities of *A. mongolicus* at different leaf growth stages based on Spearman’s correlation coefficient (|r| ≥ 0.5, *p* < 0.05). Red lines represent positive correlations and green lines represent negative correlations. (**A**) Young stages, (**B**) Mature stages, (**C**) Old1 stages, and (**D**) Old2 stages.

The interaction network among the top 200 ASVs was constructed based on an absolute Spearman’s correlation coefficient ≥ 0.5 and *p* < 0.05. Network modularity was high at all four growth stages. Total edges, average degree, density, and average clustering coefficient decreased progressively with leaf development, with the Young stage showing the highest values and the Old2 stage demonstrating the lowest. In the Old2 stage samples, these parameters decreased to 38.64%, 37.87%, 37.33%, and 81.70% of those in the Young stage, respectively. The values for the Mature and Old1 stages showed minimal variation. The proportion of positive edges remained similar across the first three stages but decreased at the Old2 stage, dropping to 80.87% of the Young stage values. The network diameter increased with leaf development, reaching its highest value at the Old2 stage, which is 2.25 times that observed at the Young stage. The percentages of negative edges and average path distance increased initially, decreased, and then rose sharply at the Old2 stage, reaching 14.67 and 2.54 times the values observed at the Young stage, respectively (Table S1). These results indicate that, with leaf development in *A. mongolicus*, the foliar endophytic bacterial network became progressively simpler, with reduced inter-node connectivity, positive co-occurrence patterns, and increased negative co-occurrence patterns (Figure 6).

### 3.4. Function of Foliar Endophytic Bacterial Communities of A. mongolicus at Different Leaf Growth Stages

**Figure 7:**
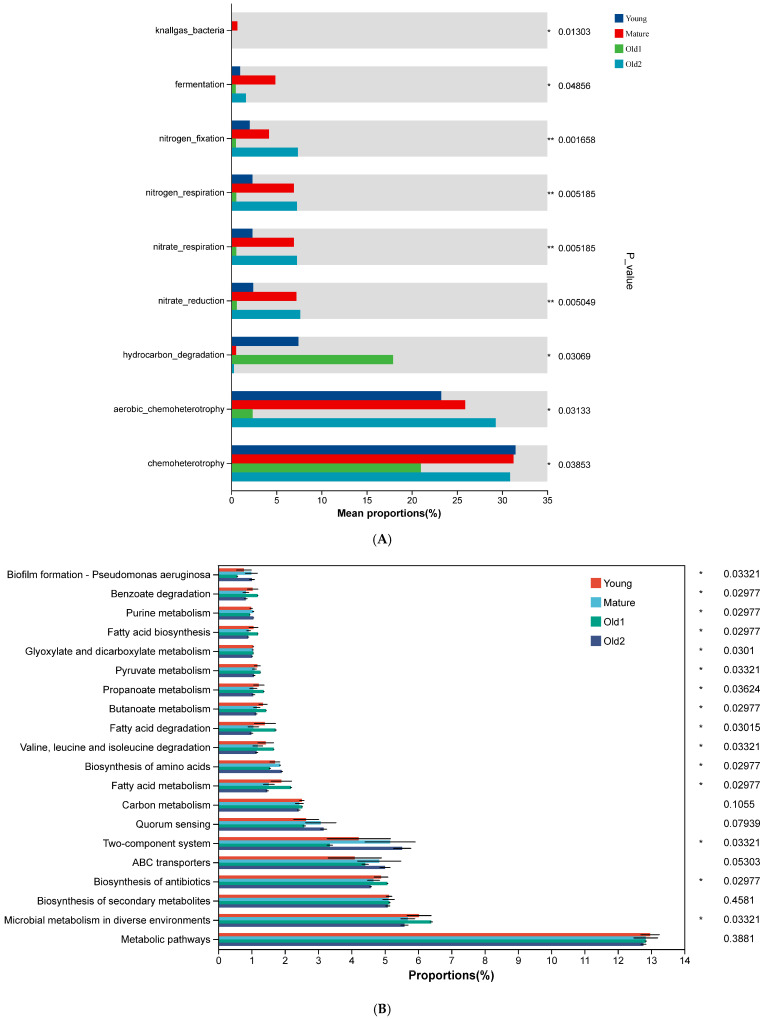
Predicted functions of foliar endophytic bacterial communities of *A. mongolicus* at different leaf growth stages: (**A**) predicted using FAPROTAX; (**B**) predicted using Tax4Fun. Asterisks denote significance: * 0.01 < *p* ≤ 0.05; ** 0.001 < *p* ≤ 0.01.

FAPROTAX was used to predict functions of foliar endophytic bacterial communities. Among the top 30 predicted functions, 9 showed significant differences across growth stages. Chemoheterotrophy and aerobic chemoheterotrophy decreased initially and then increased, with the lowest values at the Old1 stage (30% and 2.37%, respectively). Hydrocarbon degradation decreased first, and then increased before declining again, peaking at the Old1 stage (17.916%) and showing the lowest value at the Old2 stage (0.29%). Nitrate reduction, nitrate respiration, and nitrogen respiration increased initially, and then decreased and increased again, with the lowest values at the Old1 stage (0.62%, 0.57%, and 0.57%, respectively). The Mature and Old2 stages showed the highest values (7.64%, 7.29%, and 7.29%). Nitrogen fixation followed a similar trend, with the lowest value at the Old1 stage (0.51%) and the highest at the Old2 stage (7.37%) (Figure 7A).

Tax4Fun was used to predict additional functional profiles. Among the top 20 predicted functions, metabolic pathways, which showed the highest functional abundance, did not differ significantly across growth stages (*p* > 0.5). Microbial metabolism in diverse environments (second in abundance), biosynthesis of antibiotics (fourth), and fatty acid metabolism (ninth) decreased first, and then increased and subsequently decreased again, with the Old2 stage showing the lowest values (5.59%, 4.57%, and 1.47%, respectively) (*p* < 0.5). No significant differences were observed in the biosynthesis of secondary metabolites (third), ABC transporters (fifth), quorum sensing (seventh), or carbon metabolism (eighth) (*p* > 0.5). The two-component system (sixth) and biosynthesis of amino acids (tenth) functions increased initially, and then decreased and increased again, with the Old2 stage showing the highest values (5.53% and 1.91%, respectively) (*p* > 0.5) (Figure 7B).

### 3.5. Construction Process of Foliar Endophytic Bacterial Communities of A. mongolicus at Different Leaf Growth Stages

**Figure 8:**
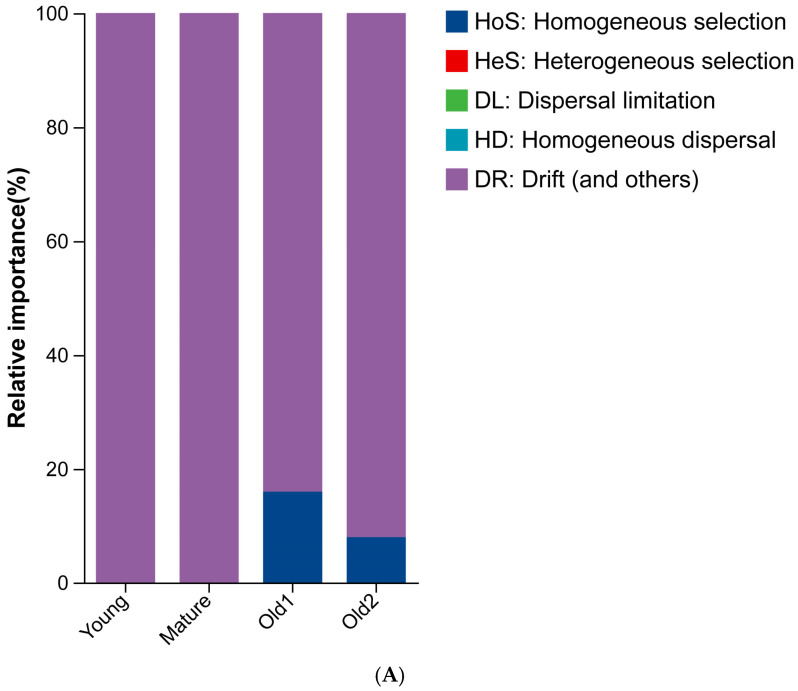

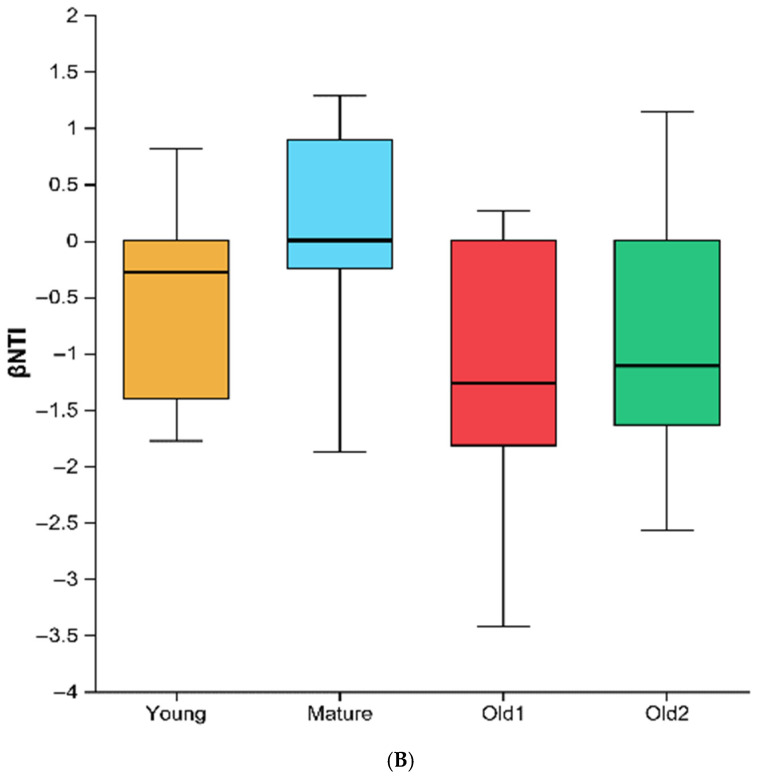
Assembly processes of foliar endophytic bacterial communities of *A. mongolicus* at different leaf growth stages: (**A**) construction process summary; (**B**) βNTI values across growth stages.

The differences in assembly processes of endophytic bacteria across the four leaf growth stages were compared. The foliar endophytic bacterial communities exhibited distinct ecological construction processes. The Young and Mature stages were mainly driven by stochastic drift (βNTI < 2 and RCBray < 0.95). The Old1 and Old2 stages were also dominated by stochastic drift but showed partial influence from homogeneous selection (βNTI < −2) (Figure 8).

### 3.6. Environmental Factors Driving Foliar Endophytic Bacterial Communities of A. mongolicus at Different Leaf Growth Stages

**Figure 9:**
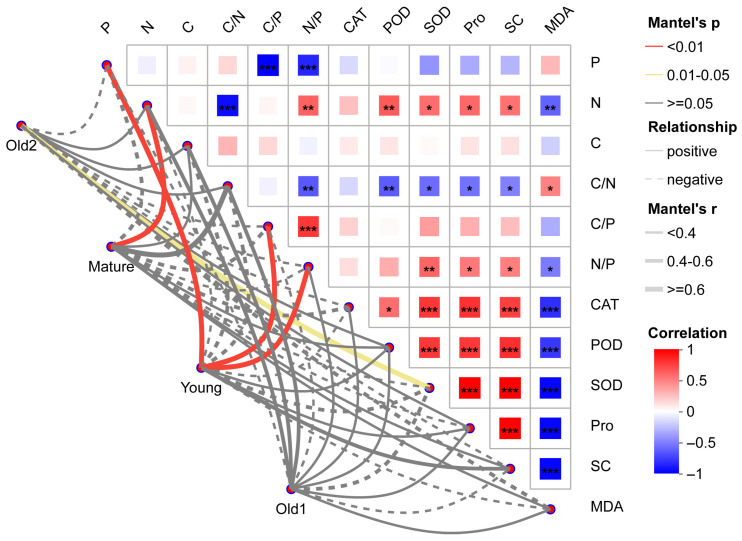
Mantel test analysis of correlations between foliar endophytic bacterial community structure and environmental factors of *A. mongolicus* at different leaf growth stages. Line thickness represents the magnitude of Mantel’s r (absolute value). Solid lines indicate positive correlations; dashed lines indicate negative correlations. Red lines indicate extremely significant correlations (*p* ≤ 0.01), yellow lines indicate significant correlations (0.01 < *p* ≤ 0.05), and gray lines indicate non-significant correlations (*p* > 0.05). The heat map shows correlations among environmental variables, with color gradients indicating positive or negative direction. Asterisks denote significance: * 0.01 < *p* ≤ 0.05; ** 0.001 < *p* ≤ 0.01, *** *p* ≤ 0.001.

Mantel test analysis of the relationships among stoichiometric characteristics (C, N, and P), physiological and biochemical parameters related to plant stress tolerance, and foliar endophytic bacterial communities showed that P, C/P, and N/P were extremely positively correlated with the Young stage (all *p* < 0.01), with Mantel’s r values of 0.65, 0.85, and 0.75, respectively. N was extremely positively correlated with the Mature stage (r = 0.95, *p* < 0.01); SOD was significantly positively correlated with the Old2 stage (r = 0.78, *p* = 0.02). No other environmental variables showed significant correlations with foliar endophytic bacterial communities (Figure 9).

## 4. Discussion

Foliar endophytic bacteria form part of the plant microenvironment and act as essential biological contributors interacting with the host. This study analyzed foliar endophytic bacterial communities in *A. mongolicus* across leaf growth stages. *A. mongolicus* is the only evergreen broad-leaved shrub in the West Ordos desert ecosystem and a relict species from the ancient Mediterranean of the Tertiary period. The results showed clear stage-dependent shifts in community structure. Community diversity increased from the Young to Mature stages, declined at the Old1 stage, and peaked at the Old2 stage. Community distribution varied markedly across growth stages, and interspecies network complexity decreased progressively.

Dominant taxa are characteristic of microbial community structure. The dominant family and genus in the Young stage samples were *Rhodococcus* (Nocardiaceae) and *Sphingomonas* (Sphingomonadaceae). In the Mature stage samples, unclassified_f__Comamonadaceae (Comamonadaceae) and *Ralstonia* (Burkholderiaceae) were dominant. In the Old1 stage samples, *Rhodococcus* (Nocardiaceae) predominated, while *Bacillus* (Bacillaceae) dominated the Old2 stage samples. In *Prunus laurocerasus*, an evergreen shrub in temperate regions, the dominant foliar endophytic genera are *Corynebacterium* (Actinobacteria), *Acinetobacter* (Gammaproteobacteria), and *Chryseobacterium* (Flavobacteria) [31]. In desert plant tissues of *Distichlis spicata* and *Pluchea absinthioides* from the Atacama Desert and *Gaultheria mucronata* and *Hieracium pilosella* from Patagonia, Chile, Pseudomonadaceae was dominant [32]. Although these plants are evergreen or desert-adapted, their endophytic bacterial distributions differ, confirming that endophytic communities are closely associated with host species [33].

Total ASV count increased with leaf development, with the Old2 stage reaching 2.42 times that of the Young stage. ASVs originated from two pathways: inheritance from the previous stage and external introduction. The number and proportion of stage-specific ASVs also increased with leaf development, whereas the proportion of ASVs shared between adjacent stages, non-adjacent stages, and across all stages decreased. This suggests a reduced contribution from the inherited pathway. Community assembly analysis showed that the Young and Mature stages were dominated by stochastic drift, while the Old1 and Old2 stages were also drift-dominated, with minor homogeneous selection influences. These results were mutually consistent.

This study also showed that although total ASVs numbers increased with leaf development, only community richness increased consistently; diversity and evenness increased initially, and then declined and rose again. Michalko et al. [31] analyzed mature one-year-old leaves of *Prunus laurocerasus* using Illumina-based 16S rRNA metabarcoding to assess shifts in endophytic bacterial communities during the seasonal transition from winter dormancy to spring growth. ASV richness increased significantly in May compared with February, March, and April. Richness trends were consistent with the present findings; however, diversity patterns differed, likely due to differences in sampling intervals between the two studies.

Factors influencing community composition were examined by assessing physiological and biochemical indicators of plant stress tolerance and stoichiometric characteristics of carbon, nitrogen, and phosphorus in leaf microenvironments. P, C/P, and N/P were significantly positively correlated with the Young stage, N with the Mature stage, and SOD with the Old2 stage. Oono et al. [34] evaluated relationships between leaf nutrient availability and endophyte diversity in *Pinus muricata* and *Vaccinium ovatum* across a soil nutrient gradient in Mendocino, California. Endophyte richness decreased in plants with higher nitrogen-to-phosphorus ratios but increased with sodium, which is potentially toxic to fungi at high concentrations. Stressed plants may exhibit low foliar nutrients or high levels of toxic compounds. Stress conditions that restrict fungal growth may increase diversity by suppressing dominant species. Zhang et al. [35] used high-throughput sequencing to analyze microbial communities in *Cinnamomum camphora* leaves at different growth stages and reported that borneol content and metabolic potential were closely linked to microbial community structure and diversity. Drivers of microbial community composition may vary depending on environmental parameters included in the analysis.

Because *A. mongolicus* is an evergreen shrub, it blossoms in the end of April (spring), sprouts new leaves in the beginning of May (spring), flourishing grows in from June (seed period), July, and August (summer) to September (Autumn), slowly grows in October (Autumn), and finally, stops growth from November, December, January, and February (winter) to next March (spring). This period is more than 5 months and spans the winter to spring periods. Therefore, we did not expect the seasons to accurately indicate the growth states of *A. mongolicus* and adopted different leaf growth stages for this study. However, the effects of seasonal environmental factors (temperature, radiation, humidity, atmospheric deposition, etc.) on foliar endophytic bacterial communities should be tested in a nested study. Moreover, functional prediction from 16S data will be utilized for validation (e.g., qPCR of functional genes, such as nif H). The potential epiphytic DNA carryover should be eliminated with PMA-qPCR.

## 5. Conclusions

This study investigated the diversity and community structure of foliar endophytic bacteria in *A. mongolicus* using Illumina 16S region (V5–V7) amplicon sequencing. Community composition varied markedly across leaf development stages. Although total ASVs numbers increased with leaf maturation, only community richness showed a consistent increase. Diversity and evenness increased initially, declined, and subsequently reached peak values at the Old2 stage. Co-occurrence networks progressively simplified. Functional predictions indicated dominant roles in carbon and nitrogen acquisition, metabolism, and environmental response. P, C/P, and N/P positively influenced foliar endophytic bacterial communities at the Young stage, N at the Mature stage, and SOD at the Old2 stage. These findings provide new insight into the diversity, composition, and function of the leaf bacterial endophytes community, particularly those related to ancient relict, evergreen, xerophytic shrubs, such as *A. mongolicus,* adapted to droughty and cold desert environments.

## Figures and Tables

**Table 1:** α-diversity index of foliar endophytic bacterial communities of *A. mongolicus* at different leaf growth stages.

	Sobs	Shannon	Simpson	Ace	Chao	Pielou	Coverage
Young	96.6 ± 19.72 ^c^	2.68 ± 0.76 ^c^	0.75 ± 0.15 ^b^	97.02 ± 17.7 ^c^	96.85 ± 19.58 ^c^	0.59 ± 0.15 ^b^	1.00 ± 0 ^a^
Mature	115.00 ± 37.78 ^c^	3.87 ± 0.19 ^b^	0.95 ± 0.02 ^a^	115.08 ± 37.95 ^c^	115.04 ± 37.87 ^c^	0.82 ± 0.07 ^a^	1.00 ± 0 ^a^
Old1	133.00 ± 44.14 ^b^	2.42 ± 0.29 ^c^	0.66 ± 0.04 ^b^	133.86 ± 44.96 ^b^	135.63 ± 47.15 ^b^	0.50 ± 0.03 ^b^	1.00 ± 0 ^a^
Old2	244.60 ± 34.25 ^a^	4.37 ± 0.18 ^a^	0.97 ± 0.01 ^a^	245.47 ± 34.17 ^a^	245.50 ± 34.31 ^a^	0.80 ± 0.02 ^a^	1.00 ± 0 ^a^

Note: Different lowercase letters indicate significant differences between leaf growth stages at the 0.05 level.

## Data Availability

Data are available from the NCBI database (https://www.ncbi.nlm.nih.gov/; accession number: PRJNA1219371; accessed on 16 April 2025).

## Supplementary Materials

The following supporting information can be downloaded at https://www.mdpi.com/article/10.3390/plants15020240/s1, Figure S1. Relative abundance and statistical differences of foliar endophytic bacterial communities of *A. mongolicus* at different leaf growth stages at the family level; Figure S2. LEfSe analysis of foliar endophytic bacterial communities of *A. mongolicus* at different leaf growth stages at the genus level; Table S1. Relevant properties of the co-occurrence network; and Table S2. Stoichiometric characteristics and physiological and biochemical parameters related to plant stress tolerance in *A. mongolicus* leaves at different growth stages.
