# Supplementary material for: Dynamics and Function of Foliar Endophytic Bacterial Communities of Ammopiptanthus mongolicus Across Different Leaf Growth Stages
Source: Plants (Basel). 2026 Jan 13;15(2):240. doi: 10.3390/plants15020240 (PMC12844642; doi:10.3390/plants15020240)
Supplement: Supplementary file 1 [file plants-15-00240-s001.zip › Supplementary Material -Figures.pdf]

Dynamics and Function of Foliar Endophytic Bacterial Communities of *Ammopiptanthus mongolicus* Across Different Leaf Growth Stages

Xue Wu, Yu Liao, Manmei Wu, Rui Yang, Qing Ma, Yuchen Wei, Jianli Liu*

(*School of Biological Science and Engineering, North Minzu University, Key Laboratory of Ecological Protection of Agro-pastoral Ecotones in the Yellow River Basin, National Ethnic Affairs Commission of the People's Republic of China, Ningxia Key Laboratory of Microbial Resources Development and Applications in Special Environment, Yinchuan, Ningxia 750021, China.*)

*Corresponding author: Jianli Liu

E-mail: LJL7523@126.com

Number of figures: 2

Supplementary Material -Figures

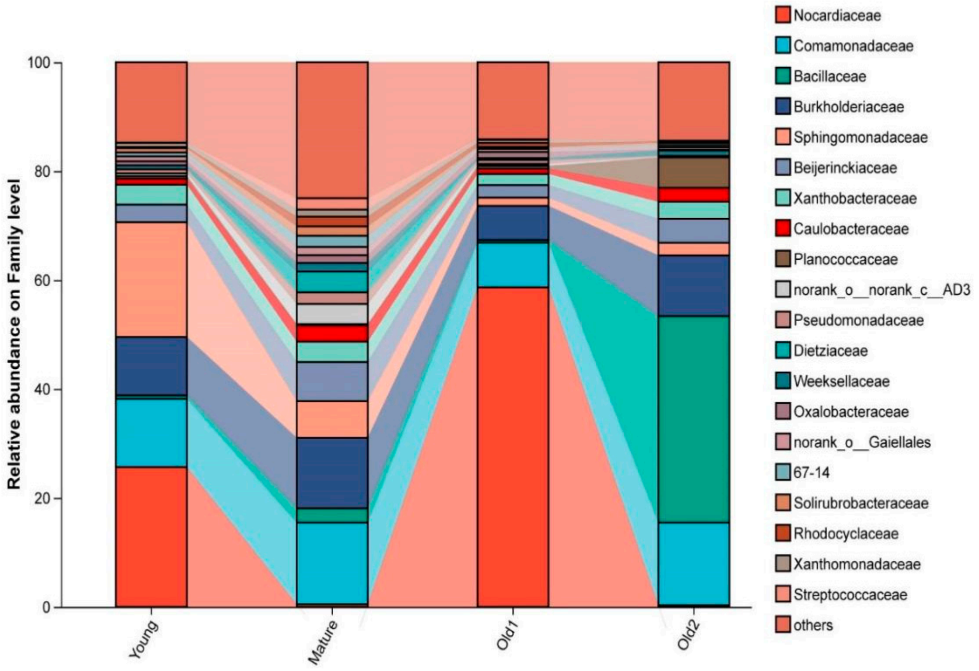

(A)

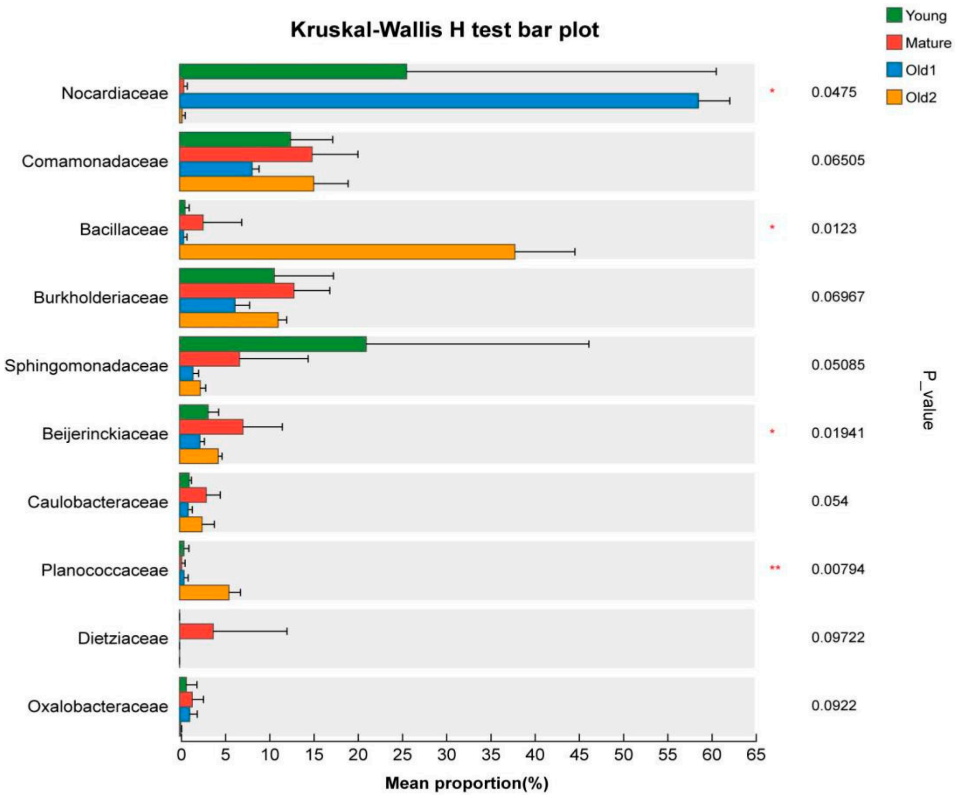

(B)

Figure S1. Relative abundance and statistical differences of foliar endophytic bacterial communities of *A. mongolicus* at different leaf growth stages at the family level. (A) relative abundance of families. (B) differences in family-level relative abundance.

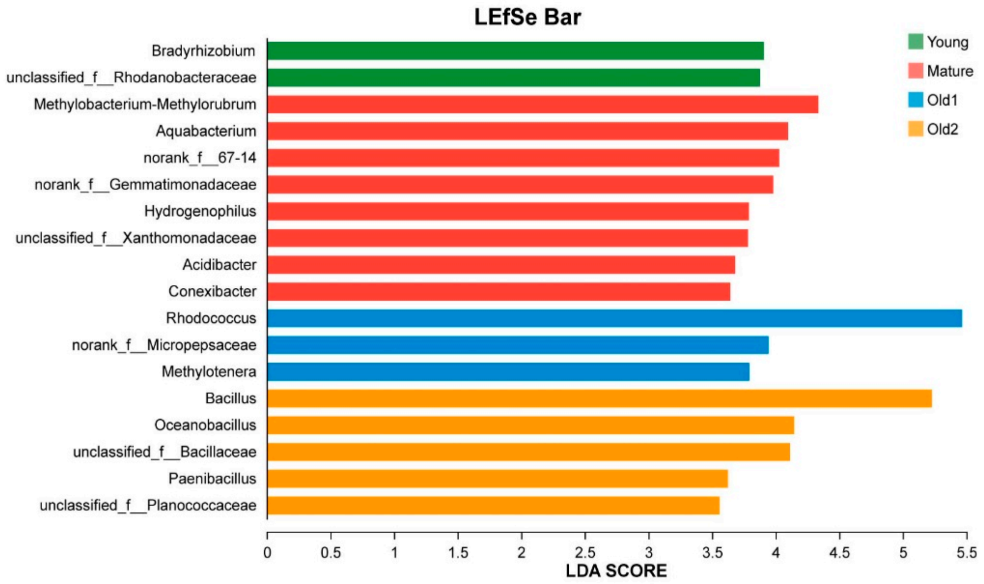

Figure S2. LEfSe analysis of foliar endophytic bacterial communities at different leaf growth stages of *A. mongolicus* at the genus level.
