# Supplementary material for: Dynamics and Function of Foliar Endophytic Bacterial Communities of Ammopiptanthus mongolicus Across Different Leaf Growth Stages
Source: Plants (Basel). 2026 Jan 13;15(2):240. doi: 10.3390/plants15020240 (PMC12844642; doi:10.3390/plants15020240)
Supplement: Supplementary file 1 [file plants-15-00240-s001.zip › Supplementary Material-Tables.pdf]

Dynamics and Function of Foliar Endophytic Bacterial Communities of *Ammopiptanthus mongolicus* Across Different Leaf Growth Stages

Xue Wu, Yu Liao, Manmei Wu, Rui Yang, Qing Ma, Yuchen Wei, Jianli Liu*

(*School of Biological Science and Engineering, North Minzu University, Key Laboratory of Ecological Protection of Agro-pastoral Ecotones in the Yellow River Basin, National Ethnic Affairs Commission of the People's Republic of China, Ningxia Key Laboratory of Microbial Resources Development and Applications in Special Environment, Yinchuan, Ningxia 750021, China.*)

*Corresponding author: Jianli Liu

E-mail: LJL7523@126.com

Number of tables: 2

Supplementary Material-Tables

Table S1. Relevant properties of the co-occurrence networks

Samples	Total edges	Negative edges	Positive edges	Average degree	Modu larity	Densi ty	Diam eter	Average clustering coefficient	Average path distance
Young	2862	39(1.38%)	2823(98.62%)	14.602	0.582	0.075	4	0.459	1.043
Mature	2129	49(2.30%)	2080(97.70%)	10.698	0.775	0.054	5	0.45	1.144
Old1	2118	41(1.93%)	2007(98.07%)	10.697	0.805	0.054	7	0.449	1.126
Old2	1106	224(20.25%)	882(79.75%)	5.53	0.78	0.028	9	0.375	2.647

Table S2. Stoichiometric characteristics and physiological and biochemical parameters related to plant stress tolerance in *A. mongolicus* leaves at different growth stages.

leaf's growth stages	C（g·kg^{-1}）	N（g·kg^{-1}）	P（g·kg^{-1}）	C: N	C:P	N:P	SC（mg·g^{-1}）	Pro（μg ·g^{-1}）	SOD（U·g^{-1}）	CAT (μmol·min^{-1}·g^{-1}）	POD（U·g^{-1}）	MDA（nmol·g^{-1}）
Young	590.83±14.33^{a}	24.10±0.22^{b}	1.24±0.08^{ab}	24.51±0.48^{a}	485.09±31.84^{ab}	19.81±1.34^{b}	4.7±0.12^{c}	212.37±3.57^{c}	534.49±11.2^{c}	13.65±0.19^{c}	6116±33.11^{d}	25.90±0.24^{b}
Mature	589.78±12.02^{a}	25.14±0.95^{b}	1.44±0.07^{a}	23.57±0.86^{a}	413.64±19.46^{b}	17.61±0.89^{b}	3.76±0.07^{d}	191.14±3.03^{d}	237.57±2.17^{d}	11.35±0.16^{d}	7396±32.50^{c}	30.91±0.28^{a}
Old1	589.32±9.84^{a}	24.63±0.57^{b}	1.40±0.03^{ab}	24.01±0.95^{a}	420.89±8.64^{ab}	17.62±0.68^{b}	8.66±0.07^{b}	241.09±2.76^{b}	812.78±6.82^{b}	31.39±0.18^{a}	8988±58.86^{b}	9.03±0.18^{c}
Old2	596.05±4.91^{a}	28.59±0.83^{a}	1.21±0.08^{b}	20.92±0.56^{b}	500.27±32.95^{a}	23.95±1.63^{a}	11.69±0.07^{a}	303.34±5.43^{a}	892.03±10.09^{a}	28.29±0.22^{b}	9572±45.87^{a}	7.69±0.17^{d}
